# Extended Driving Impairs Nocturnal Driving Performances

**DOI:** 10.1371/journal.pone.0003493

**Published:** 2008-10-22

**Authors:** Patricia Sagaspe, Jacques Taillard, Torbjorn Åkerstedt, Virginie Bayon, Stéphane Espié, Guillaume Chaumet, Bernard Bioulac, Pierre Philip

**Affiliations:** 1 GENPPHASS, CHU Pellegrin, Bordeaux, France; 2 MSIS, INRETS, Paris, France; 3 CNRS UMR-5227, Bordeaux, France; 4 Karolinska Sleep Institute, Stockholm, Sweden; 5 Hôpital Hôtel-Dieu, Paris, France; 6 Université Bordeaux 2, Bordeaux, France; University of Sydney, Australia

## Abstract

Though fatigue and sleepiness at the wheel are well-known risk factors for traffic accidents, many drivers combine extended driving and sleep deprivation. Fatigue-related accidents occur mainly at night but there is no experimental data available to determine if the duration of prior driving affects driving performance at night. Participants drove in 3 nocturnal driving sessions (3–5am, 1–5am and 9pm–5am) on open highway. Fourteen young healthy men (mean age [±SD] = 23.4 [±1.7] years) participated Inappropriate line crossings (ILC) in the last hour of driving of each session, sleep variables, self-perceived fatigue and sleepiness were measured. Compared to the short (3–5am) driving session, the incidence rate ratio of inappropriate line crossings increased by 2.6 (95% CI, 1.1 to 6.0; P<.05) for the intermediate (1–5am) driving session and by 4.0 (CI, 1.7 to 9.4; P<.001) for the long (9pm–5am) driving session. Compared to the reference session (9–10pm), the incidence rate ratio of inappropriate line crossings were 6.0 (95% CI, 2.3 to 15.5; P<.001), 15.4 (CI, 4.6 to 51.5; P<.001) and 24.3 (CI, 7.4 to 79.5; P<.001), respectively, for the three different durations of driving. Self-rated fatigue and sleepiness scores were both positively correlated to driving impairment in the intermediate and long duration sessions (P<.05) and increased significantly during the nocturnal driving sessions compared to the reference session (P<.01). At night, extended driving impairs driving performances and therefore should be limited.

## Introduction

Though fatigue and sleepiness at the wheel are well-known risk factors for traffic accidents [1]–[5], many drivers combine sleep deprivation and driving [6], [7]. This dangerous behaviour can be related to economic rewards in professional drivers [8] or to socio-cultural factors in vacationers [7], [9]. We showed in past studies that young drivers were highly exposed to sleep curtailment during long distance driving [7], [9].

Because of these conflicts between physiological needs and social or professional activities [10], [11], understanding the human limits of fatigue and sleep deprivation are becoming key issues in accident prevention.

Sleepiness can be defined as a difficulty in remaining awake even while carrying out activities [12]. This symptom is related to circadian and homeostatic influences. The biological clock generates and maintains chronobiological rhythms which control sleep and wakefulness. During the daytime, there is a short duration drop of vigilance in the mid-afternoon followed by a very alert period towards the end of the afternoon [13]. Extended time awake and/or sleep restriction will increase sleep pressure and generate cumulative sleepiness [14], [15] which is known to impair neurobehavioral functioning [16]–[19]. Interaction between these two regulatory processes induces a non-linear evolution of sleepiness over time. Sleepiness is eliminated by a period of sleep.

Fatigue is a gradual and cumulative process associated with a disinclination towards effort, eventually resulting in reduced performance efficiency [20]. It has been described in driving episodes which require sustained attention for long periods of time [21]. Fatigue is eliminated by a period of rest. Several studies show increased risk as a function of time at the wheel. Most show that it takes around nine or ten hours before accident proneness starts to rise [22]. Kaneko and Jovanis (1992) [23], who investigated the driving patterns of truck drivers and found that night time driving was the most relevant factor, also showed that the risk of having an accident increased once the driving time had gone beyond nine hours. However, these studies usually include confounders like increased time awake (due to late driving), curtailed prior sleep (due to early starts), or driving during the circadian low. In a study by Philip et al. (2005) [24], it was demonstrated that 10 hours of driving did not affect driving performance, but prior sleep loss did. This suggests a lack of effect of the duration of driving. However, there was no control situation of short duration driving included so the effect of duration of driving per se could not be evaluated. Furthermore, this study was carried out during the day while fatigue-related accidents occur predominantly at night [25]–[27]. The study also contained 15 minute breaks every two hours, which may have prevented accumulation of fatigue.

Against the background above the present study was designed to be the first one to use a dose-response design of duration of driving while controlling for effects of prior time awake, prior sleep time, and time of day. The particular durations selected were 2, 4 and 8 hours representing common durations of driving [7] and the experiment was scheduled as night driving.

## Methods

### Participants

Fourteen young healthy men (mean age [±SD] = 23.4 [±1.7] years, range 21–25 years, mean yearly driving distance [±SD] = 14,250 [±4660] km) were recruited by advertisement in the hospital and university and provided written informed consent. Subjects were paid 300 € for the whole experiment. The study protocol was approved by the local ethics committee (Comité de Protection des Personnes Sud-ouest et Outre-mer III de Bordeaux).

### Inclusion criteria

All participants underwent a clinical interview with a sleep specialist and a nocturnal polygraphy to rule out any sleep disorders. Because sleep duration and sleep efficiency are crucial in sleep-restriction protocols, we used actimeters (Actiwatch®, Cambridge Neurotechnology, Cambridge, UK) to quantify our volunteers' sleep duration. This device monitors body movements and allows calculation of mean nocturnal sleep episodes and of nocturnal awakenings.

Time in bed was also computed as the time difference between going to bed in the evening and getting up in the morning. Sleep efficiency was calculated as the ratio of time asleep to the time in bed, in percentage. To rule out any sleep-wake schedule disorders, each participant was monitored for 7 days before being included in the study. Participants were included if they had a mean sleep efficiency of at least 85% during the 3 days of recordings.

### Study design

The study uses a cross-over balanced design, with all participants having 3 nocturnal driving sessions on the open road after normal sleep. All subjects drove for 2, 4 and 8 hours (3–5 am, 1–5 am and 9 pm–5 am). At least 5 days elapsed between 2 sessions.

### Sleep Schedules

The participants were instructed to maintain a regular sleep-wake schedule and were monitored by actimetry during the 3 days preceding each experimental session. Participants were instructed to sleep between 11 pm to 7 am the night before driving. No stimulant of any kind was allowed during the study. Subjects were prevented from sleeping by technicians in the laboratory from 7 am to the start of the driving session.

### Driving session

**Figure 1:**
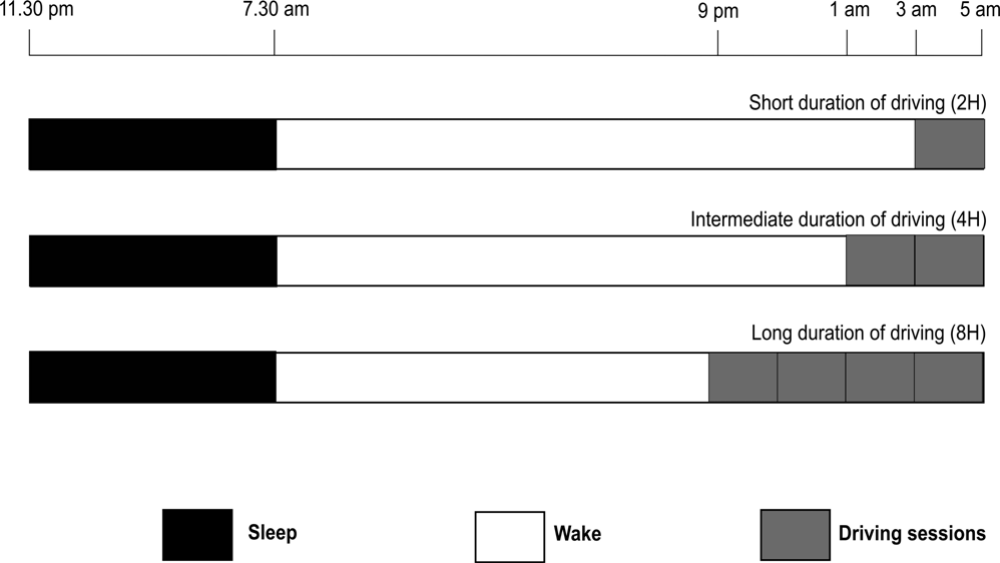
Design of the protocol representing the sleep-wake period and the duration of each nocturnal driving session (short, intermediate and long durations of driving).

Subjects drove the short session of 230 km (143 miles) from 3 to 5 am (2 hours of driving), the intermediate session of 460 km (286 miles) from 1 to 5 am (4 hours of driving), and the long session of 920 km (572 miles) from 9 pm to 5 am (8 hours of driving). The same section of 115 km highway was driven in one direction and then back for the short condition (230 km). For the intermediate condition, two such laps were driven and for the long conditions, four laps were driven (see Figure 1).

Driving took place on a straight, two-lane highway on weekdays under light traffic conditions, and in fair weather. Subjects were instructed to maintain a constant speed (130 kph; 80 mph), to drive in the center of the lane, and not to cross the painted lines separating the lanes, except to pass a slower vehicle. During the whole experiment, a professional driving instructor monitored the driving speed and was ready to take control of the car (equipped with dual controls) if needed. No verbal communication was allowed between the drivers and co-pilots unless a specific instruction had to be given (i.e. stop to the next rest area). In order to allow sufficient rest for the co-pilots, three instructors alternated every two hours of driving during the 8 hours driving condition.

The car used for the experiment was equipped with a Siemens® video system which measures and registers 10 times/sec. the lateral position of the vehicle from the right lateral lane marker of the road [28].

At the beginning and at the end of each lap, subjects were asked to rate their fatigue on a 100 mm visual analogical scale (VAS) (“Describe how fatigued you are now”) ranging between “not at all tired” and “very tired”, and the Karolinska Sleepiness Scale (KSS) [29]. KSS was also completed in the middle of each lap. The scale ranges from 1 to 9 (“very alert” to “very sleepy, fighting sleep, an effort to keep awake”).

### Outcome measures

Several studies have shown that impaired daytime alertness induces lateral deviations during driving [19], [24], [30], [31] and sleep related accidents frequently occur, with a single car driving off the road and hitting an obstacle with no reaction from the driver [1]. Therefore we selected inappropriate line crossings as our main outcome criterion to quantify driving impairment after sleep restriction. An inappropriate line crossing was counted each time the car crossed one of the lateral highway lane markers except during a passing maneuver or some other necessary driving action. All deviations related to traffic interference were excluded from the analyses to concentrate on the remaining line crossings, presumably related to driver's status. Our secondary outcome measures were self-rated fatigue and sleepiness (Karolinska Sleepiness Scale), and sleep parameters during the subsequent sleep.

### Data processing and analysis

Actimetric data are reported as means and standard deviation (SD).

In order to avoid effects of start-up in the short session we only compared the ILC values for the last hour of each drive, instead of the possible two hours. We then used the first hour of driving (9–10 pm) of the 9 pm–5 am driving session in order to provide the reader with a reference obtained under presumably alert driving.

The number of inappropriate line crossings were analyzed using negative binomial regression in Stata, version 9.0 (Stata Corp., College Station, USA), using number of line crossings in the last hour of driving per participant as dependent variables and driving sessions (9–10 pm, 3–5 am, 1–5 am and 9 pm–5 am) as determinants clustered on participants. We reported comparisons of ILC between conditions as incidence rate ratios (IRR) with 95% confidence intervals (CI).

The effects of nocturnal driving sessions on self-rated fatigue and sleepiness were evaluated by Friedman and Wilcoxon non-parametric test. Non parametric correlations (Rho Spearman) were computed between fatigue and sleepiness scores and driving performance (Inappropriate Line Crossing) in the last hour of driving for each driving sessions.

## Results

### Sleep Variables

Participants slept for a mean of 428±33 min (sleep efficiency, 91±6%) during the night before the short (3–5 am) driving session, for a mean of 412±46 min (sleep efficiency, 88±10%) during the night before the intermediate (1–5 am) driving session and for a mean of 431±48 min (sleep efficiency, 91±9%) during the night before the long (9 pm–5 am driving) session. The difference was not significant.

### Driving performance

**Figure 2:**
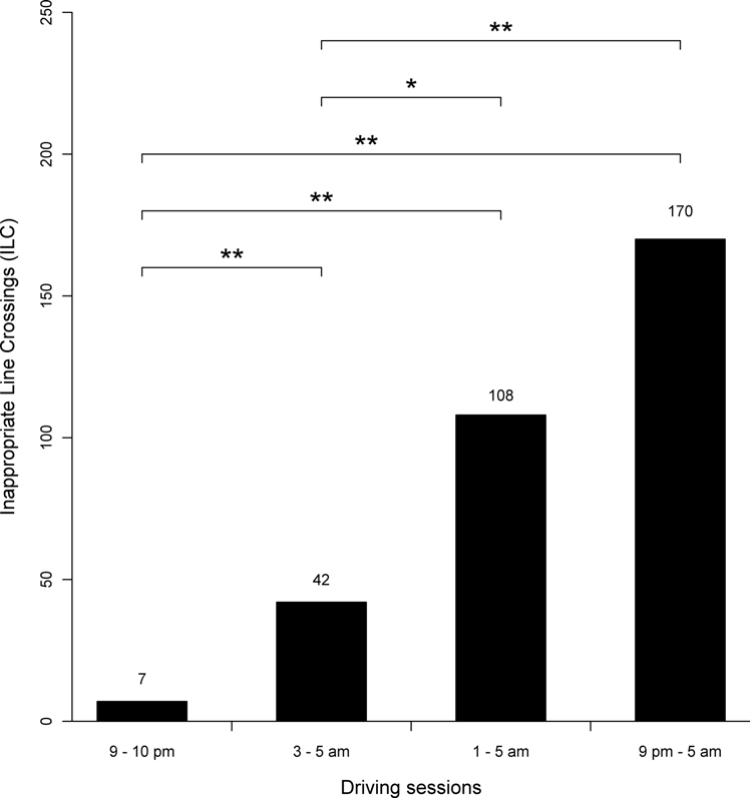
Cumulative number of inappropriate line crossings (ILC) for the 14 subjects in the last hour of the 3 nocturnal driving sessions (short, intermediate and long durations of driving), as well as the ILC for the reference drive (9–10 pm of the long drive). Statistical analyses refer to Incidence rate ratios (IRR) with 95% confidence intervals (CI) using the short drive and the 9–10 pm drive as reference (See section results). * P<.05. ** P<.001.

There were significant differences among the 2, 4 and 8 hours driving sessions (see Figure 2). Compared to the short driving session, the IRR of ILC increased by 2.6 (95% CI, 1.1 to 6.0; P<.05) in the last hour of the intermediate session, and by 4.0 (CI, 1.7 to 9.4; P<.001) in the long session 9 pm–5 am driving session (See Figure 2).

There was also a significant increase of inappropriate line crossings between the reference driving session (9–10 pm) and the last hour of the 3 nocturnal driving sessions (see Figure 2). Compared to the reference period (9–10 pm), the incidence rate ratio (IRR) of inappropriate line crossings (ILC) was 6.0 (95% CI, 2.3 to 15.5; P<.001) for the short session, 15.4 (CI, 4.6 to 51.5; P<.001) for the intermediate session and 24.3 (CI, 7.4 to 79.5; P<.001) for the long session (See Figure 2).

### Self-Perception of Fatigue

After the long drive, fatigue scores were significantly higher than after the intermediate drive (Wilcoxon rank sum test = −2.989, P<.01) (See Table 1).

**Table 1:** Fatigue (VAS) scores after the last hour of driving (Mean±SD) and Karolinska Sleepiness Scale (KSS) scores before the last hour of driving (Mean±SD) in the reference session and in the 3 nocturnal driving sessions.

	Reference	Nocturnal driving sessions		
		3–5 am	1–5 am	9 pm–5 am
**Fatigue (VAS)**	40.0 (±13.6)	65.1 * (±15.9)	66.8 * (±18.2)	75.0 * □ (±18.2)
**KSS**	2.6 (±1.2)	6.8 * (±1.8)	7.4 ** (±1.3)	8.0 ** (±1.4)

The asterisks refer to significant differences between the 3 nocturnal driving sessions (short, intermediate and long durations of driving) and the reference. The square refers to a significant difference between 9 pm–5 am and 1–5 am sessions.

* P<.01.

** P<.001.

□ P<.01.

Fatigue (Visual analog scale) after the last hour of driving differed across the 4 driving sessions (Friedman test = 24.928, P<.001). Fatigue significantly differed between the reference (11 pm) and the 3 nocturnal driving sessions (respectively, Wilcoxon rank sum test for the short drive = −2.731, P<.01; for the intermediate drive = −3.234, P<.01, and for the long drive = −3.298, P<.01).

Self-rated fatigue after the last hour of driving correlated with the number of inappropriate line crossing in the intermediate (Rho = 0.527, P<.05) and in the long drive (Rho = 0.478, P = .08 (tendency)).

### Self-Perception of Sleepiness

Sleepiness before the last hour of driving differed significantly from the reference (9 pm) and the three nocturnal sessions with different durations of prior driving (For the short drive, Wilcoxon rank sum test reached = −3.077, P<.01; for the intermediate drive = −3.306, P<.001 and for the long drive = −3.317, P<.001). Before the last hour of driving, sleepiness was identical in the 3 nocturnal driving sessions (See Table 1).

Self-rated sleepiness before the last hour of driving correlated with the number of ILC in the intermediate driving session (Rho = 0.611, P<.05) and in the long driving session (Rho = 0.608, P<.05).

### Self-Perception of Fatigue and Self-Perception of Sleepiness

Self-rated fatigue after the last hour of driving did not correlate with the self-rated sleepiness before the last hour of driving in the short (Rho = 0.244, NS) and intermediate drive (Rho = 0.288, NS), but correlated in the reference (Rho = 0.603, P<.05) and long drive (Rho = 0.672, P<.01).

## Discussion

Our results clearly show a major impact of duration of nocturnal driving on the number of inappropriate line crossings. This was established using inappropriate line crossings as the main outcome variable. This choice was based on several factors apart from face validity. Thus, epidemiologic findings show that 65% of sleep-related accidents occur after an inappropriate line crossing [32]. Several studies [24], [30], [33], [34] have shown that impaired daytime alertness induces lateral deviations on the road during driving, and sleep-related accidents frequently occur with one car driving off the road and hitting an obstacle without appropriate reaction from the driver [32], [35]. Interestingly, the number of inappropriate line crossings has been related to the risk of accidents [36], subjects reporting one near-miss accident in the last 3 years have an increased risk of 1.13 of being involved in an accident within the past year, compared to normals. Similarly, subjects having 4 near-misses have an increased risk of 1.87 of being involved in an accident within the past year.

The findings of a considerable effect of duration of driving on driving performance give a new dimension to the impact of fatigue on driving performance [24]. Apparently, the duration of driving is an independent factor that impairs nocturnal driving performance beyond that of sleep homeostatic and circadian factors. In this study, we controlled for such influences by using the same timing (last hour) for the comparison of all driving sessions. Thus, all three nocturnal sessions had the same homeostatic and chronobiological pressure and therefore differences of performance between the three driving sessions can only be explained by the increase of fatigue generated by a longer driving session.

The present results differ from our prior study [24] which did not find that driving impairment increased across 10 hours but daytime driving obviously differs from night time driving. The reason for the discrepancy may be, either that the effect of duration is not present during day driving, or that the breaks in the former study neutralized any effects of duration of driving. We previously showed that caffeine improves nocturnal driving performance in both young and middle-aged participants and that aging does not reduce the effectiveness of the response to caffeine [37]. In contrast, after a short nap, younger drivers improved their performances much more than did middle-aged ones. It would be worthwhile investigating the differential effects of a break, taking a nap or caffeine on sleepiness/fatigue and driving performance.

Using the first hour (9–10 pm) as reference it was shown that the last hour of the three main driving conditions strongly differed from the reference. This mainly shows that the outcome ILC and ratings of fatigue and sleepiness could respond to high levels of alertness. The results also suggest that circadian and homeostatic factors appear to be more important than the duration of driving [4], [24]. This should, however, be subjected to a separate study.

With respect to self-rated fatigue there was a clear effect of the duration of the drive when intermediate and long duration were compared. A difference was not found, however, between the latter and the short drive, which was unexpected, but could be a coincidence. There may have been inadequate power to detect an effect and a larger sample size may have found a significant difference in sleepiness and fatigue scales between the different driving conditions. Subjective sleepiness before the last hour of driving did not differ between any of the main conditions. The reason may be an inability to differentiate sleepiness between three already high levels. Also here a larger sample would have resulted in significant effects.

Interestingly, both fatigue and sleepiness correlated with driving impairment but sleepiness seems to give an earlier signal than fatigue regarding driving risk. Apparently, the drivers' own appreciation of their state of fatigue/sleepiness is at least as good in real life driving situations as in simulated driving [38].

There are several limitations of the present study. Real life studies, while necessary for ecological validity do provide less control than laboratory studies. In the present study, however, there does not seem to have occurred any major problems that may have affected the results systematically. In addition, the number of subjects was modest and generalizations should be made with caution. However, the results seem robust. Further research should investigate if similar findings apply to professional drivers, which have a higher expected frequency of nocturnal driving.

In summary, the interaction of duration of driving at night with circadian clock time impairs driving performance and therefore road safety. Long drives during the day are not the same as long drives during the night. This lends support to the legislative work on driving regulations, which tend to focus strongly on time at the wheel as a tool for improving safety. It also suggests that the public should be advised to limit the distance driven at night. However, one should also emphasize that homeostatic and circadian factors seem more important.
